# Supplementary material for: Splice-Junction-Based Mapping of Alternative Isoforms in the Human Proteome
Source: Cell Rep. Author manuscript; Available in PMC 2020 Jan 15. (PMC6961840; doi:10.1016/j.celrep.2019.11.026)
Supplement: 2 [file NIHMS1546469-supplement-2.zip › DF1/PXD010154/Esophagus/Esophagus_3_TNXB_MGPLSVVIVTVAR.pdf]

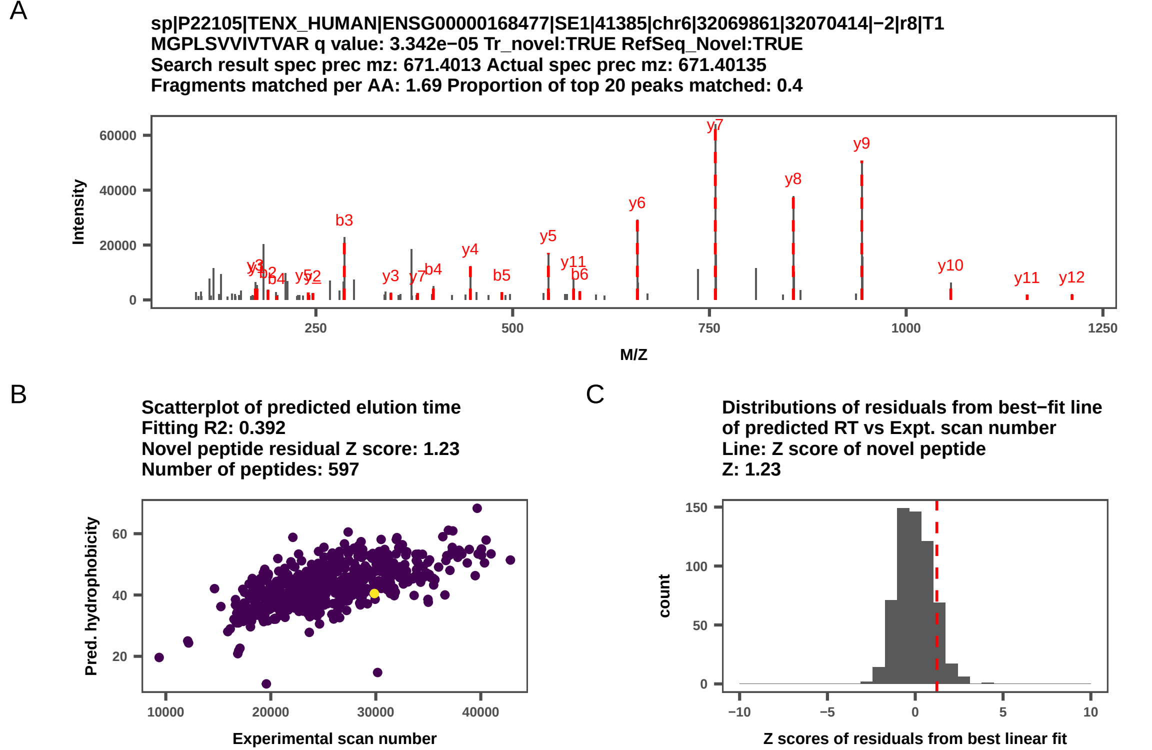
A
sp|P22105|TENX_HUMAN|ENSG00000168477|SE1|41385|chr6|32069861|32070414|−2|r8|T1
MGPLSVVIVTVAR q value: 3.342e−05 Tr_novel:TRUE RefSeq_Novel:TRUE
Search result spec prec mz: 671.4013 Actual spec prec mz: 671.40135
Fragments matched per AA: 1.69 Proportion of top 20 peaks matched: 0.4
Intensity
60000
40000
20000
0
250
500
750
1000
1250
M/Z
b3
y3
y4
y5
y6
y7
y8
y9
y10
y11
y12
b4
b5
b6
B
Scatterplot of predicted elution time
Fitting R2: 0.392
Novel peptide residual Z score: 1.23
Number of peptides: 597
Pred. hydrophobicity
60
40
20
10000
20000
30000
40000
Experimental scan number
C
Distributions of residuals from best−fit line
of predicted RT vs Expt. scan number
Line: Z score of novel peptide
Z: 1.23
count
150
100
50
0
−10
−5
0
5
10
Z scores of residuals from best linear fit
